# Isolation and microencapsulation of *Lactobacillus* spp. from corn silage for probiotic application

**Published:** 2010-06

**Authors:** R Kasra – Kermanshahi, J Fooladi, S Peymanfar

**Affiliations:** 1 Department of Biological Science, Alzahra University, Vanak, Tehran, Iran

**Keywords:** *Lactobacillus*, silage, microencapsulation, chitosan, alginate

## Abstract

**Background and Objectives:**

Probiotics including strains of *Lactobacillus* spp. are living microorganisms including which are beneficial to human and animals health. In this study, *Lactobacillus* has been isolated from corn silage in a cold region of Iran by anaerobic culture.

**Materials and Methods:**

The bacteriological and biochemical standard methods were used for identification and phenotypic characterization of isolated organism. To increase the stability of organism in the environment, we used microencapsulation technique using stabilizer polymers (Alginate and Chitosan).

**Results:**

The isolated *Lactobacillus* spp. was able to ferment tested carbohydrates and grow at 10°C–50°C. Using microencapsulation, the stability and survival of this bacterium increased.

**Conclusion:**

microencapsulation of lactic acid bacteria with alginate and chitosan coating offers an effective way of delivering viable bacterial cells to the colon and maintaining their survival during refrigerated storage.

## INTRODUCTION

Probiotics are living bacteria and fungi that are administered in order to provide health benefits for the host (1). Ensiling is a method of preservation for large masses of moist crops from aerobic and anaerobic deterioration caused by certain microorganisms such as Clostridia, Enterobacteria, moulds and *Listeria monocytogenes*. It is based on lactic acid fermentation under anaerobic conditions whereby lactic acid bacteria (LAB) convert water soluble carbohydrates into organic acid, mainly to lactic acid. As a result, the pH decreases and the moist of crop is preserved. The aim of this study was to isolate probiotics from silage and to stabilize it for survival under extremely harsh environment and subsequently feeding the livestock. The properties of desirable probiotic bacterium for silage include: growth at 50°C, tolerance to acidic environment (pH 4) and bile salts, disability in Dextran production but capability in consumption of penthose and hexhose (2). Microencapsulation techniques have been successfully used to enhance dairy fermentation for the production of concentrated lactic acid bacteria and to improve the survival of microorganisms in dairy products and mayonnaise (12, 13). Among the encapsulation devices, microencapsulation in calcium alginate microparticles has been widely used for the immobilization of lactic acid bacteria owing to its ease of handling, nontoxic nature, and low cost (3). It is a method that preserve bacteria from detrimental factors of environments such as high acidity (low pH), bile salts (4, 5), molecular oxygen in case of obligatory anaerobic microbes, bacteriophages and chemical as well as antimicrobial agents. Alginate, a cheap and non-toxic material to the body, is used for the encapsulation of viable bacterial cells. It is a linear heteropolysaccharide extracted from different types of algae, with two structural units consisting of D-mannuronic and L-guluronic acids. Calcium alginate has been widely used for the encapsulation of lactic acid and probiotic bacteria. Chitosan is another material for encapsulation. Chitosan is a linear polysaccharide with negative charge arising from its amine groups which are obtained by deacetylation of chitin. It is soluble at pHs lower than 6. Chitosan has been used for coating of alginate capsules (6, 7). The prebiotic potential of chitosan was studied. Chitosan has showed antibacterial activities (8). The objective of this study was to investigate the effect of chitosan-coated alginate microparticles on the survival of isolated *Lactobacilli* from silage.

## MATERIALS AND METHODS

**Isolation and Identification of *Lactobacillus* spp.** Corn silage was collected from a cold region. Silage was added into 90 ml of saline solution (0.85%, pH 7) and shaken by hand. From the suspension, 10^-1^ to 10^-3^ serial dilution was prepared. From the first and last dilutions, 100 µl of suspension were added into the tubes containing 10 µl MRS broth (Merck, Germany) for enrichment. Then tubes were incubated in an anaerobic jar at 37°C (9). After one day incubation, tubes that contain turbidity were spread on the plates containing the MRS agar. After 2 days incubation at 37°C, pure culture was obtained. Lactic acid bateria (LAB) was detected by the presence of yellowish colony. One colony was selected for identification. The biochemical standard tests were used for identification of isolates organisms and these include: Gram staining, catalase reaction, carbohydrates fermentation (glucose, galactose, lactose, manitol and fructose), growth at 10°C and 50°C in MRS broth and hydrolysis of arginine (10). The MRS broth base containing 0.5% different sugars and 0.004% purple bromo cresol and lacking glucose and meat extract was used and for carbohydrate fermetatation test. Same medium containing arginine (0.3%) and sodium citrate (0.2%) was used to test the hydolysis of arginine.

**Analysis of probiotic properties**. Acid tolerance was studied by using acidic PBS (0.03 g KH_2_ PO_4_, 0.18 g Na_2_ HPO_4_- 2H_2_O, 0.18 g NaCl for 20 ml PBS) and neutral PBS. For this purpose, bacterium was grown in 50 ml of MRS broth at 37°C for 40 hours. After 2 days, this suspension was centrifuged at 3000 rpm for 15 minutes. The pellets was re-suspended into the 20 ml acidic PBS (pH 2.5) and anaerobically incubated at 37°C. Then suspension was centrifuged at 5000 rpm for 30 minutes. The pellet was re-suspended into the 20 ml neutral PBS and centrifuged at 3000 rpm, 15 minutes at 4°C. Then the pellet was re-suspended into few saline solution and 100µl from suspension was added into 9.9ml saline solution and well shaken. Serial dilution was performed until 10^−8^ dilution spread on MRS agar petridish. From 10^−6^, 10^−8^ dilutions, 100 µl of suspension was spread on MRS agar petridishs. The petridishes were incubated anaerobically at 37°C for 2 days and the colonies were counted. This work was performed at pHs of 3.5, 4.5, 5.5, 6.5 and the results were investigated (9). For bile salts tolerance tests, the bacterium was cultivated into the MRS broth containing 0.3% deoxycholate sodium (bile salt). From cultivation time to later 8 hours, per 0.5 hours, absorbance was measured by the spectronic spectrophotometric (OD_600_). MRS broth without bile salt was used as blank in spectrophotometry (9). The bacterium was cultivated into a Gibson semisolid medium and incubated anaerobically at 37°C to determine whether they are recognizing heterofermentative or homofermentative. The Gibson medium contained 2.5 g meat extract, 50 g Glucose, 100 ml tomato juice, 800 ml free fat milk,10 ml from% 0.4 MnSO_4_, nutrient agar for 200 ml and distilled water were added for 1000 ml medium (11).

**Preparation of chitosan–coated alginate microparticles loaded with *Lactobacillus.*** The procedure explained by Gaserod *et al* (14) was used for micoencapsulation. Sodium alginate, Chitosan and Xanthan gum were supplied by Sigma (Sigma Aldrich, St. Louis, MO). Calcium chloride, tween 20 (polyoxyethylene sorbtan monolaurate), Glycerol and KCl – HCl buffer solution of pH 2.0 were purchase from Merck (Germany).

The alginate mixture was prepared by adding% 2 (w/v),% 5.5 (w/v) MRS broth,% 5 (v/v) glycerol,% 0.26 (w/v) Xanthan gum,% 0.1 (v/v) Tween 20 and% 20 (v/v) cell suspension into distilled water and then mixed together.

*Lactobacillus* that isolated from silage, was cultivated into the MRS broth for 40 hours at 37°C anaerobically. After 2 days, cells were harvested by centrifugation at 1500 g for 15 minutes at 25°C and washed twice with sterile saline solution. The pellet was re-suspended into the MRS broth and was divided into two parts: One part was used for microencapsulation and the other was used as free cells without capsule (for control) (12). This work was performed for *Lactobacillus plantarum* ATCC 8014 as control. The mixture was infused with a magnetic bar and was placed through a insulin syringe and sprayed through a flask containing 1000 ml of 0.5 M CaCl_2_ solution under gentle stirring with a magnetic bar. The divalent calcium ions cross-linked the droplets of sodium alginate to form alginate microparticles. The microparticles formed were allowed to harden in CaCl_2_ solution for 15 minutes and filtered through two layers of unsterile filter paper. The filtered alginate microparticles were rinsed twice with sterile distilled water and then transferred to a solution of chitosan. Chitosan (0.4 g) was dissolved in 90 ml distilled water acidified with 0.4 ml of glacial acetic acid to achieve a final concentration of 4 g/L. The pH was then adjusted to between 5.7 and 6.0 by adding 1 mol/L NaOH. The microparticles were stirred gently with a magnetic bar for 15 min to evenly coat the surface of the alginate microparticles. The chitosan solution and alginate solution were autoclaved at 110°C for 5–7 minutes. Then washed beads were immersed in 100 ml of chitosan solution and stirred gently with a magnetic bar for 15 minutes to coat the surface of the alginate microparticles. The resulting chitosan – coated alginate microparticles were again separated by paper filteration and rinsed twice with distilled water (13). The microparticles were stored in a sterile petridish, and placed at 4°C, 8°C and 25°C. To study the survival of bacteria, microparticles were obtained after 24 h and they liquefied by adding 1 g of bead to 99 ml sterile%1 sodium citrate solution (pH 6.0). The Serial dilution was prepared and pour-plating 1 mL on MRS agar. The plates were incubated at 37°C for 48 h.

The colony forming units of microorganisms were counted to assess survival. Average survival of *Lactobacilli* was determined from three separately diluted and plated samples. The experiments were replicated three times. Therefore, the results are presented in mean survival rate+standard deviation. This work was performed for 1 week and 1 month later (14).

## RESULTS

**Morphological and biochemical properties.** The isolated bacterium was gram-positive, catalase-negative and heterofermentative bacillium with yellowish, mocoid, rounded colonies.

**Fig. 1:**
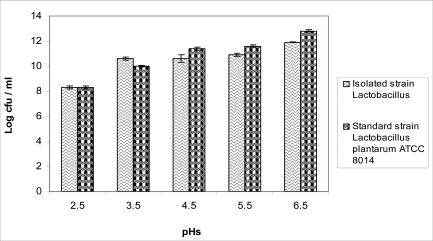
Comparison of acid tolerance between isolated *Lactobacillus* and type strain *Lactobacilllus plantarum* ATCC 8014 at 37°C. Results are indicated by the vertical bars. All mean survival rates were significantly different (p<0.05).

**Fig. 2:**
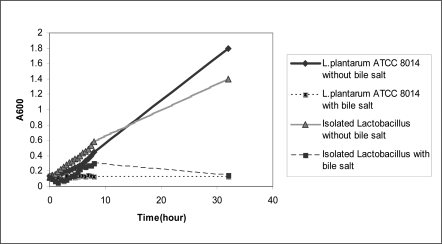
Comparison of bile salt tolerance between isolated *Lactobacillus*, type strain *Lactobacillus plantarum* ATCC 8014. All mean survival rates were significantly different (p<0.05).

The isolated *Lactobacillus* grew at 10°C–50°C, hydrolyzed arginine and produced NH_3_. The production of NH_3_ was detected by Nesler reagent. The acid tolerance pattern and count of bacterium in different acidic pHs is shown in Fig. 1. The tolerance of the organisms to bile salt tolerance assay is shown in Fig. 2.

**Morphological analysis of microcapsules.** The size of microcapsules was 1–2 mm (8°C, 25°C) and it did not alter after 1 month but their size was reduced after storing at 4°C. They were contaminated with fungi after one month storage at 25°C.

**Fig. 3:**
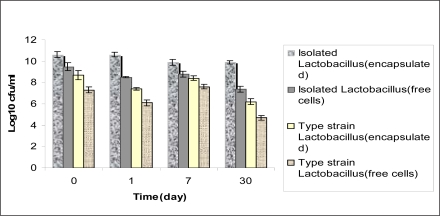
Comparison of viability between of free and encapsulated isolated *Lactobacillus*, type strain *Lactobacillus plantarum* ATCC 8014 at 8°C Results are indicated by the vertical bars (p<0.05)

**Release of microparticles and study of cells viability.** After releasing of the cells from microcapsules, the cells were enumerated by spectrophotometric and plate count methods (Fig. 3).

## DISCUSSION

The mechanism of effectiveness of a probiotic is closely associated with the properties of the production of strains. *Lactobacillus* are often found in association with plant materials, dairy products and as the dominant microbial population on forage crops and silages (15). Many studies (16, 17), have reported that the inoculation of forage with homofermentative *Lactobacilli* such as *L. casei, L. plantarum* have beneficial effects on promoting lactic acid fermentation and improving silage quality. However, the heterofermentative *Weissella* and *Leuconostocs* did not improve silage quality and may have caused some fermentation loss. In this study, we isolated *Lactobacillus spp.* from silage that was heterofermentative and acid tolerant bacterium. Results showed that acid tolerance of isolated *Lactobacillus* varied at different pHs. The isolated *Lactobacillus* was bile salt intolerant. Microencapsulation techniques have been successfully used to enhance dairy fermentation for the production of concentrated lactic acid bacteria and to improve the survival of microorganisms in dairy products (18, 19). Studies of Lee and et al have showed that both free and microencapsulated cells showed similar stabilities during 4 weeks of storage at 4°C (12). Microencapsulation of isolated bacterium with alginate and chitosan showed higher stability than free cell culture during 1 month. Between encapsulated isolated *Lactobacillus* and type strain *Lactobacillus* was low difference (p<0.05). Several studies showed that the survival of microencapsulated bacteria was improved in alginate microparticles in compared with the stored nonencapsulated bacteria (12–14, 18, 19). Krasaekoopt et al. (13) reported that probiotic bacteria loaded in chitosan-coated alginate microparticles showed higher storage stability than free cell culture. Future studies need to be carried out in order to monitor the effect of microencapsulation on bacteria in the gut, using animal models, as well as studying other parameters such as the initial cell numbers and bead size.
